# Regional variations in the diet of the South Polar Skua (*Stercorarius maccormicki*) in the Ross Sea region, Antarctica

**DOI:** 10.1371/journal.pone.0341112

**Published:** 2026-02-09

**Authors:** Jihee Kim, Youmin Kim, Jong-U. Kim, Younggeun Oh, Jeong-Hoon Kim

**Affiliations:** 1 Division of Life Sciences, Korea Polar Research Institute, Incheon, Republic of Korea; 2 Department of Agriculture, Forestry, and Bioresources, Seoul National University, Seoul, Republic of Korea; 3 Department of Life Sciences, Incheon National University, Incheon, Republic of Korea; Tshwane University of Technology, SOUTH AFRICA

## Abstract

The South Polar Skua (*Stercorarius maccormicki*) is an opportunistic feeder, predator, and scavenger widely distributed in the coastal ecosystems of Antarctica. However, although some studies have explored its foraging behavior, many aspects, including regional variations, remain insufficiently understood. Thus, this study aimed to characterize regional variations in the dietary composition of South Polar Skuas breeding at sites along the Northern Victoria Land coast in the Ross Sea, where environmental conditions vary markedly among colonies. The dietary composition and foraging characteristics of the South Polar Skua populations in the Ross Sea region were investigated by analyzing stable carbon (δ¹³C) and nitrogen (δ¹⁵N) isotopes in blood samples. The values were then used to quantify isotopic niches and estimate dietary contributions. Results revealed significant regional variations in the dietary composition of skuas. Compared with skuas at other sites, skuas at Cape Möbius showed higher δ¹³C values, suggesting a greater reliance on fish and the placenta or carcasses of Weddell seals for food. While δ¹⁵N values did not significantly differ among skuas at various sites, skuas at Cape Möbius and Cape Washington had higher values than those at other sites, suggesting access to higher trophic-level prey. Site-specific dietary contributions were revealed, with Adélie Penguin eggs and fish dominating the diet at Cape Hallett and Inexpressible Island, whereas Emperor Penguin eggs and fish were more prominent at Cape Washington. Our findings demonstrate that the species exhibits site-specific foraging patterns shaped by local prey availability. These results provide novel insights into the trophic ecology of this top predator and contribute to a broader understanding of the spatial dietary variation in Antarctic seabirds.

## Introduction

South Polar Skuas (*Stercorarius maccormicki*) are long-lived apex predatory seabirds with a circumpolar distribution during the breeding season [1]. As a generalist and opportunistic feeder, this species consumes a wide variety of prey, including fish, krill, and the eggs or chicks of other seabirds [2–4]. They also scavenge on penguin carrion and seal remains, including carcasses and placentas [4]. However, their dietary composition varies substantially depending on the environment and local prey availability in breeding sites [3–5]. This study examines regional variation in the diet of South Polar Skuas as an aspect of their foraging ecology, focusing on differences among breeding sites in the Ross Sea region. Understanding how South Polar Skua responds to local environmental variability is critical for assessing the ecological flexibility of top predators in Antarctic food webs. Spatial differences in prey use may reflect adaptive foraging strategies in response to fluctuating resource availability, including changes in prey density across breeding sites and seasonal shifts during the breeding period [6,7]. These patterns may provide insights into the degree of dietary specialization within heterogeneous ecosystems. We therefore hypothesize that the functional role of South Polar Skuas as scavengers is strongly influenced by the ecological context and prey availability surrounding their colonies in the Ross Sea.

To date, relatively few studies have examined the diet of this species across different regions of Antarctica. In areas where South Polar Skuas breed sympatrically with Brown Skuas (*Stercorarius antarcticus lonnbergi*), their dietary divergence appears to be shaped by interspecific competition. Generally, the former primarily forage at sea on fish, whereas the latter rely heavily on terrestrial prey such as penguin eggs and chicks [8–11]. By contrast, at Deception Island, South Polar Skuas consume more adult penguin carcasses, whereas Brown Skuas feed predominantly on chicks [12]. In the absence of interspecific competition, South Polar Skuas also exhibit site-specific dietary preferences. For instance, on Ross Island, skuas at Cape Bird, where Adélie Penguins (*Pygoscelis adeliae*) breed in large numbers, primarily consume penguin-derived prey. Meanwhile, individuals at Cape Evans, approximately 12 km from the nearest penguin colony, show significantly reduced reliance on penguins [7]. Similarly, at Ardery Island, where Adélie Penguins are absent, skuas feed on seabird species in proportion to their local breeding abundance [3]. These findings suggest that the opportunistic foraging behavior of South Polar Skuas enables them to exploit the most numerous and readily accessible prey species in each habitat.

An estimated 50% of the global South Polar Skua population is thought to reside in the Ross Sea region [7,13]. Along the coast of Northern Victoria Land within this region, skuas breed at several sites that differ markedly in local prey availability and biological communities. Such environmental heterogeneity is expected to influence skua foraging behavior and shape their trophic interactions, potentially leading to regional variation in dietary composition and isotopic niche breadth [14,15].

Traditional methods for assessing seabird diets, such as the morphological analyses of feces, regurgitated pellets, or prey under a microscope, provide valuable short-term insights but are limited in resolution and biased toward hard remains [3,9,12]. These methods may underestimate the contribution of soft-bodied or fully digested prey and are often constrained by sampling effort and taxonomic expertise [16–21]. By contrast, stable isotope analysis of carbon (δ¹³C) and nitrogen (δ¹⁵N) offers a powerful tool to evaluate long-term dietary patterns and trophic positions, as isotopic signatures integrate feeding information over extended periods and can distinguish prey from different trophic levels and habitat origins [19,20,22]. In general, the turnover time of stable carbon and nitrogen isotopes in avian blood plasma is approximately 3 days, whereas that of red blood cells ranges from 2 to 4 weeks [23]. In Great Skuas (*Stercorarius skua*), red blood cell turnover is approximately 15.7 days [24]. These turnover rates indicate that isotope values from different tissues reflect dietary intake over distinct temporal windows, which is an important consideration when interpreting results from stable isotope analyses.

Although several studies have investigated the diet of South Polar Skuas in different parts of Antarctica, comparative studies within the Ross Sea, particularly along the coast of Northern Victoria Land, remain limited. By comparing multiple breeding sites that differ in local prey availability, this study provides a regional-scale assessment of dietary variation in South Polar Skuas based on stable isotope analysis. The primary objective of this study is to describe and compare the diets of South Polar Skuas from different breeding populations along the northern coast of Victoria Land in relation to the ecological characteristics of each site.

## Materials and methods

### Study area and sample collection

**Fig 1:**
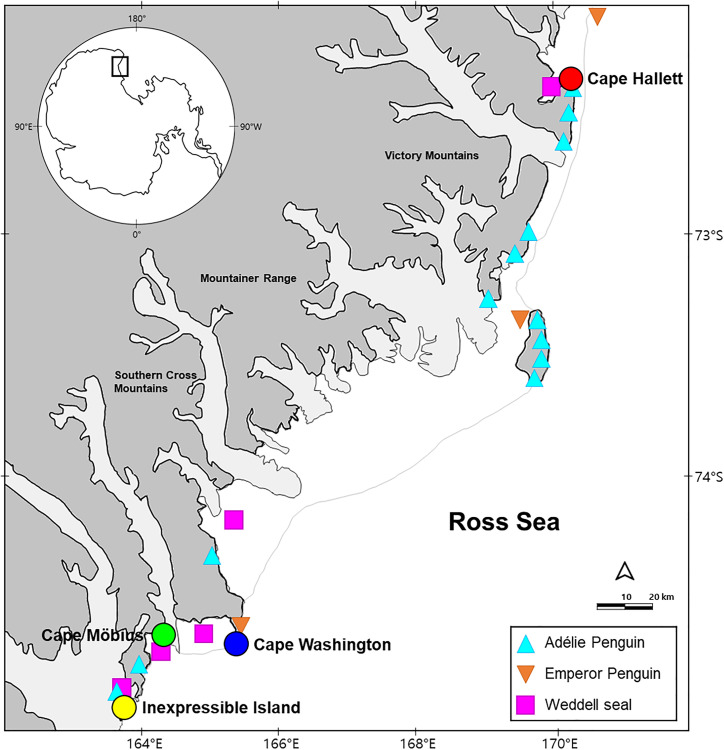
Location of the study area. The study sites are located along the cost of Northern Victoria Land, Antarctica. Sampling sites are marked with colored circles (red: Cape Hallett; blue: Cape Washington; green: Cape Möbius; and yellow: Inexpressible Island), and breeding sites of potential prey species of the South Polar Skua are indicated with triangles. This map was generated by the authors using publicly available map data (coastlines and breeding sites of prey) from the Korea Polar Research Institute (www.kopri.re.kr). The figure was prepared in Microsoft PowerPoint and is published under a CC BY 4.0 license.

This study was conducted at four breeding sites located in the Northern Victoria Land coast, Ross Sea region, Antarctica (Fig 1; 72°20′–75°50′ S, 163°30′–171°20′ E). Cape Hallett (72º19′ S, 170º13′ E) is a key breeding site in the Ross Sea region, supporting approximately 40,000 pairs of Adélie Penguins. Because the breeding seasons of Adélie Penguins and South Polar Skuas overlap, penguin-derived prey are predictably abundant for skuas during the breeding period [4,13]. Nearby, Weddell seals breed in Edisto Inlet (72°20′ S, 170°05′ E), while Cape Roget (71°59′17″ S, 170°36′04″ E) hosts a colony of Emperor Penguins. By early November, the landfast ice extends more than 10 km from the shore. Cape Washington (74°39′ S, 165°25′ E) hosts a breeding colony of approximately 20,000 pairs of Emperor Penguins. The site is also located near Weddell seal haul-out areas, such as Silverfish Bay, and is relatively close to open marine habitats where fish and krill may be accessible. Other nearby breeding sites include Edmonson Point (74°20′ S, 165°8′ E) for Adélie Penguins, although the high density of skuas relative to Adélie Penguins makes this area less accessible for skuas from Cape Washington [25]. By early November, the landfast ice is more narrowly distributed, extending less than 10 km from the shore. Cape Möbius, located near the Jang Bogo Station, is another important breeding site, with Weddell seals breeding in the nearby Terra Nova Bay. An Adélie Penguin colony is present at Adélie Cove, approximately 17 km away, and tracking data indicate that skuas breeding at Cape Möbius also visit Cape Washington (unpublished data). By early November, the fast ice extends broadly, covering over 30 km. Inexpressible Island supports a breeding colony of approximately 20,000 pairs of Adélie Penguins, while nearby Hells Gate is a breeding site for Weddell seals. A polynya exists in the area, and by early November, the ice cover is almost nonexistent, extending less than 2 km from the shore. At all breeding sites, approximately 100 pairs of South Polar Skuas breed with population fluctuations depending on the breeding seasons [26].

Skuas were captured using a bownet (8-feet 1-inch net, Northwood Falconary) and were attracted by replica eggs placed in nests or by prey items. Blood samples were collected from the skuas during the incubation and hatching periods, between November and December 2021. Whole blood (ca. 0.1 mL) was collected from the brachial vein of adult skua using disposable syringes within 10 min of capture. During handling, each bird was placed in a bird holding bag that covered their eyes to reduce visual stimulation, which helped calm them and minimize struggling. All individuals were handled gently, sampling was completed quickly, and they were released immediately at the capture site. No anesthesia or analgesia was used, as the procedure was minimally invasive, and no birds were sacrificed during this study. All procedures were conducted with the approval of the Institutional Animal Care and Use Committee of the Korea Polar Research Institute (KACC2101−003), and the study was carried out in accordance with the SCAR Code of Conduct for the Use of Animals for Scientific Purposes in Antarctica.

Of the 41 whole blood samples of adult skuas, 8 were collected at Cape Hallett, 13 at Cape Washington, 14 at Cape Möbius, and 6 at Inexpressible Island. Considering that no recent reference stable isotope values were available for this species from the region, we sampled Emerald rockcod (*Trematomus bernacchii*), a potential food source for skua, by fishing at Jang Bogo Station. All of the samples were transported to the laboratory stored at −20 °C, and freeze-dried before stable isotope analysis.

### Stable isotope analysis

Stable isotope ratios change during metabolic processes, as lighter isotopes are preferentially lost and heavier isotopes are retained, resulting in consumer tissues being isotopically heavier than their prey [27]. δ¹³C is typically close to that of the diet and thus reflects the origin of primary producers, serving as an indicator of energy sources [27,28]. By contrast, δ¹⁵N typically increases by approximately 3‰-5‰ per trophic level and is widely used as an indicator of trophic position [27–29]. In the present study, whole blood was used for the analysis, and sampling was conducted during the incubation and hatching periods. Thus, the isotopic values are expected to reflect the pre-laying and early incubation stages, given the turnover time of whole blood. The ratios of nitrogen (δ^15^N) and carbon isotopes (δ^13^C) in the samples were analyzed using an elemental analyzer combustion continuous-flow stable isotope ratio mass spectrometer at the National Instrumentation Center for Environmental Management at Seoul National University. Lipid removal was not performed because of the negligible lipid content in blood [24,30,31]. The stable isotope values were calculated using the following equation:

δX (‰) = [(R_sample_/R_standard_) - 1] × 1,000,

where X represents either ^15^N or ^13^C, R denotes the ratio of heavy to light isotopes (^15^N/^14^N or ^13^C/^12^C), and R_standard_ refers to the international reference standard (atmospheric nitrogen for δ^15^N and Vienna Pee Dee Belemnite for δ^13^C). Repeated measurements indicated that standard deviations for this study were <0.1‰ for δ^13^C and <0.2‰ for δ^15^N.

### Isotope model and statistical methods

Differences in nitrogen and carbon isotopic values among skuas at each breeding site were assessed using one-way analysis of variance (ANOVA), followed by post-hoc comparisons using Tukey’s honest significant difference (HSD) test (α = 0.05). The nitrogen and carbon isotopic niches of skuas in different habitats were determined using Stable Isotope Bayesian Ellipses in R (SIBER) [14]. This method provides an estimate of dietary variation and serves as an indicator of their foraging strategy, reflecting the degree to which individuals exhibit generalist or specialist feeding behaviors [14,15]. Two isotopic niche metrics were calculated: the total hull area, also known as the Layman metric, and standard ellipse area corrected for small sample size (SEAc), both with 40% confidence intervals. The proportional contributions of each potential prey item to the diet of skuas were estimated using the Bayesian stable isotope mixing model MixSIAR (Mixing Models for Stable Isotope Analysis in R), which incorporates isotopic variation and uncertainty [32,33]. All statistical analyses were performed in R [34].

We identified potential food sources at each site through previous studies and field surveys (Table 1). Skuas feed on krill either through direct predation or via secondary ingestion by preying on krill-consuming species such as penguins or fish [37]. According to the draft Research and Monitoring Plan for Ross Sea Region Marine Protected Area by CCAMLR, Antarctic krill (*Euphausia superba*) are abundant in the open waters north of the Ross Sea, particularly around Cape Hallett, whereas Ice krill (*E. crystallorophias*) are commonly found further south and coastal regions [20,38,39]. Consequently, ice krill are a potential food source for skuas at the three other sites. Fish also comprise a substantial portion of the skua diet in various regions [8–11]. In the Ross Sea region, skuas were observed regurgitating Antarctic silverfish (*Pleuragramma antarctica*) and Emerald rockcod (*T. bernacchii*) to feed their chicks. This behavior was also recorded by motion-sensor cameras installed at skua nests (S1 Fig). Accordingly, the stable isotope values for the fish category were represented by the average of two species. To assess the potential for skuas to prey on penguins at each site, we considered the breeding distributions of Adélie and Emperor Penguins. Adélie Penguins breed near Cape Hallett, Cape Möbius, and Inexpressible Island, whereas Emperor Penguins breed near Cape Hallett, Cape Washington, and Cape Möbius [40]. Therefore, we considered the likelihood of Adélie Penguins serving as a major food source for skuas at Cape Washington and Emperor Penguins at Inexpressible Island to be low. Skuas prey not only on penguin chicks but also on a substantial number of eggs [4,41]. Given the marked difference in the stable isotope values between chicks and eggs, these food sources were treated separately in the analysis. Skuas also feed on Weddell seal remains, including carcasses, placentas, and feces [4] (S1 Fig). For the MixSIAR modeling, we used previously reported stable isotope values of potential prey items (Table 1). The trophic enrichment factors (TEFs) applied for skuas were 1.1‰ for δ^13^C and 2.8‰ for δ^15^N, which were based on TEF values for blood of the Great Skua (*S. skua*) [24], as species-specific TEFs are not currently available for the South Polar Skua.

**Table 1:** Stable isotope values (δ^13^C and δ^15^N (mean ± SD)) of potential food sources at four sites in the Ross Sea region: Cape Hallett (CH), Cape Washington (CW), Cape Möbius (CM), and Inexpressible Island (II). Presence and absence of the species at each site are denoted by “O” and “X,” respectively.

Food sources	δ^13^C (‰)	δ^15^N (‰)	CH	CW	CM	II	Reference
**Antarctic krill**	−27.41 ± 0.57	3.65 ± 0.58	O	X	X	X	[20]
**Ice krill**	−24.52 ± 0.77	6.12 ± 0.51	X	O	O	O	[20]
**Fish**	−24.79 ± 0.97	10.48 ± 0.89	O	O	O	O	This study
Antarctic silverfish	−25.00 ± 0.80	10.30 ± 0.80	O	O	O	O	[35]
Emerald rockcod	−23.66 ± 1.24	11.74 ± 0.22	O	O	O	O	This study
**Adélie Penguin**	−25.80 ± 0.23	7.10 ± 0.33	O	X	O	O	[36]
**Adélie Penguin egg**	−27.50 ± 0.33	9.20 ± 1.11	O	X	O	O	[36]
**Emperor Penguin**	−25.00 ± 0.75	13.40 ± 0.43	O	O	O	X	[36]
**Emperor Penguin egg**	−28.30 ± 0.65	12.30 ± 1.10	O	O	O	X	[36]
**Weddell seal**	−21.30 ± 0.70	15.70 ± 0.60	O	O	O	O	[19]

## Results

### Stable isotope values

**Fig 2:**
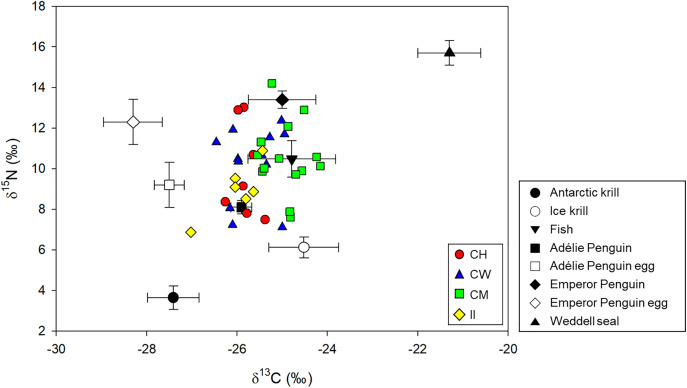
Stable isotope values in whole blood samples of South Polar Skuas at each site (CH: Cape Hallett; CW: Cape Washington; CM: Cape Möbius; and II: Inexpressible Island). Values were adjusted using trophic enrichment factors (1.1‰ for δ^13^C and 2.8‰ for δ^15^N), shown alongside those of their potential food sources.

**Fig 3:**
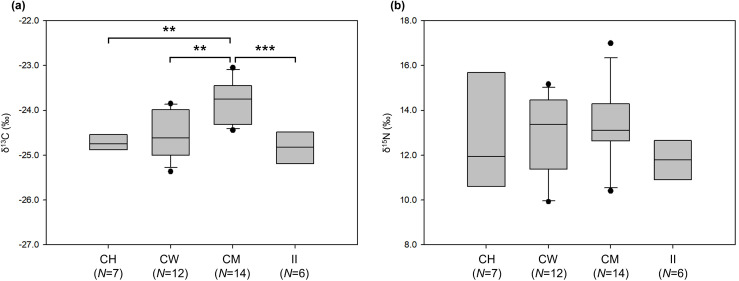
Boxplots of stable isotope values (δ¹³C and δ¹⁵N) in whole blood samples of South Polar Skuas. (a) δ^13^C of individuals (n = 39) at each breeding site. (b) δ^15^N of individuals (n = 39) at each site (CH: Cape Hallett; CW: Cape Washington; CM: Cape Möbius; and II: Inexpressible Island). Asterisks indicate significance levels from Tukey’s post-hoc pairwise comparisons between sites: ***p ≤ 0.001. **p ≤ 0.01.

Of the 41 samples in total, two samples that deviated significantly were excluded as outliers (δ^13^C: −22.11‰, δ^15^N: 10.78‰ from Cape Hallett, and δ^13^C: −24.77‰, δ^15^N: 21.67‰ from Cape Washington), and the remaining data were used in all subsequent statistical analyses and modeling. Stable isotope ratios in 39 individuals of skua ranged from 9.68‰ to 17.00‰ for δ^15^N (mean ± SD: 12.89‰ ± 1.83‰) and from −25.92‰ to −23.05‰ for δ^13^C (mean ± SD: −24.37‰ ± 0.63‰) (S1 File). The TEF-corrected values with the mean ± SD values of their potential food sources (Table 1) are presented in Fig 2. The δ¹³C values significantly varied among breeding sites (Fig 3), with post-hoc pairwise comparisons revealing differences between Cape Hallett and Cape Möbius (*p* = 0.001), Cape Möbius and Cape Washington (*p* = 0.002), and Cape Möbius and Inexpressible Island (*p* = 3.0 × 10^−4^). By contrast, although the mean δ^15^N was highest at Cape Washington, followed by Cape Möbius, Cape Hallett, and Inexpressible Island, the differences in δ^15^N among the breeding sites were not significant (one-way ANOVA, Tukey’s HSD test, *F*_*3,35*_ = 1.075, *p* = 0.372).

### Isotopic niches

**Fig 4:**
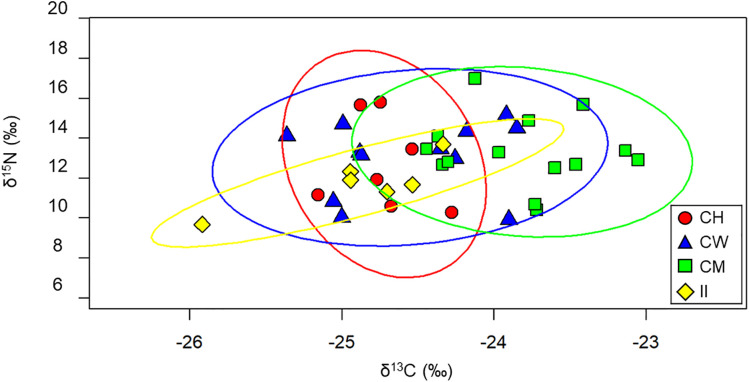
Isotopic niches (CI = 0.95) for South Polar Skuas (n = 39) from four breeding sites. CH: Cape Hallett (n = 7); CW: Cape Washington (n = 12); CM: Cape Möbius (n = 14); and II: Inexpressible Island (n = 6).

Isotopic niches showed extensive overlap among the four sites, with no clear separation, as indicated by the 95% confidence ellipse (Fig 4). The largest isotopic niche was observed in skuas from Cape Washington (SEAc: 3.29), followed by Cape Möbius (SEAc: 2.69), Cape Hallett (SEAc: 2.34), and Inexpressible Island (SEAc: 1.53).

### Diet contribution

**Fig 5:**
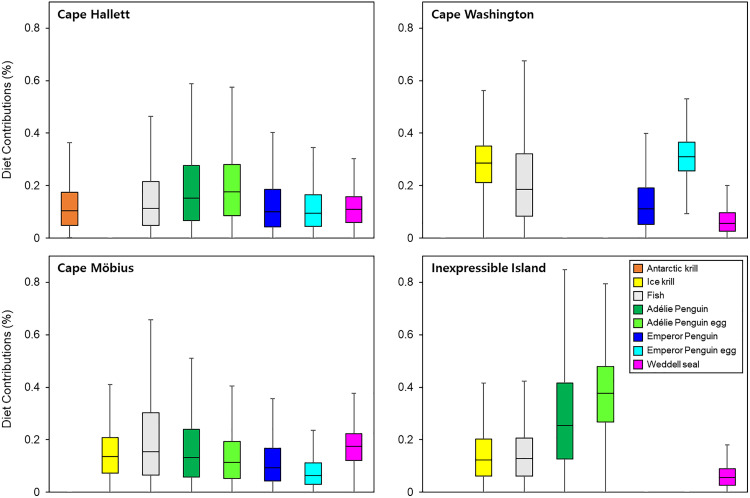
Estimated proportional contributions of potential food sources to the diet of South Polar Skuas at each breeding site.

The diet of skuas at Cape Hallett was estimated to be primarily composed of Adélie Penguin (eggs, 0.19‰ ± 0.13‰; birds, 0.19‰ ± 0.15‰, mean proportion ± SD), with fish comprising 0.15‰ ± 0.13‰ (Fig 5). In the three other sites, fish represented a consistent dietary component: 0.21‰ ± 0.15‰ at Cape Washington, 0.20‰ ± 0.16‰ at Cape Möbius, and 0.15‰ ± 0.11‰ at Inexpressible Island. At Cape Washington, Emperor Penguin eggs (0.31‰ ± 0.08‰) made the highest contribution to the diet of skuas, followed by ice krill (0.28‰ ± 0.10‰). The diet of skuas at Cape Möbius included notable contributions from fish, Weddell seal (0.17‰ ± 0.07‰), and Adélie Penguin (0.16‰ ± 0.13‰). At Inexpressible Island, Adélie Penguin eggs were a predominant food item (0.37‰ ± 0.15‰) in the skua diet, with Adélie Penguins contributing (0.28‰ ± 0.19‰).

## Discussion

This study investigated regional variations in the diets of South Polar Skuas in the Ross Sea region, Antarctica by using stable isotope analysis. The results demonstrate that skuas adopt opportunistic foraging strategies, predominantly targeting the most abundant or accessible prey at each site. These findings highlight the ecological flexibility of South Polar Skuas in adapting their foraging strategies to local prey availability, a vital trait for survival in the resource-limited Antarctic ecosystem.

The δ¹³C values of skuas at Cape Möbius were significantly higher than those at the other study sites (Fig 3a). This enrichment likely reflects a reduced reliance on krill and krill-dependent Adélie Penguins, along with greater consumption of fish and the carcasses or placentas of piscivorous Weddell seals (Fig 5). By contrast, δ^15^N values did not differ significantly among sites (Fig 3b), possibly due to high individual-level variation and the limited sample size. However, the δ^15^N values at Cape Möbius, located near the Weddell seal breeding site, and at Cape Washington, adjacent to an Emperor Penguin breeding colony, were higher than those at Inexpressible Island and Cape Hallett, both of which are associated with Adélie Penguin colonies. These spatial patterns suggest that skuas at Cape Möbius and Cape Washington have greater access to prey from higher trophic levels than those at the other sites. The two isotopic outliers excluded from the analysis may reflect unusual short-term dietary events, such as opportunistic scavenging or the consumption of atypical prey shortly before sampling [22,24,29].

Food availability in Antarctica is generally limited, and skuas are therefore expected to exhibit an opportunistic foraging strategy [4,5], leading to a broad isotopic niche across sites [14,15] (Fig 4). In addition to prey availability, isotopic niche breadth may be influenced by intraspecific competition associated with colony size, which can promote dietary diversification [42]. However, because the number of breeding pairs of South Polar Skuas was similar across the study sites [26], this factor is unlikely to explain the observed variation in niche breadth. We collected whole blood samples between early November and mid-December, which correspond to the incubation and hatching periods. This timing coincides with the incubation period of Adélie Penguins and the chick-rearing period of Emperor Penguins [43], when dead eggs are often present on sea ice. It also overlaps with the post-parturition period of Weddell seals [19], during which skuas were observed consuming placentas (unpublished data). These temporal overlaps likely influenced prey availability and, consequently, the isotopic signatures observed in this study. Nevertheless, the foraging patterns of skuas vary among sites, reflecting site-specific dietary differences. In colonies of large Adélie Penguins, such as Cape Hallett and Inexpressible Island, skuas primarily secure prey by hunting chicks or stealing eggs, which provides a relatively stable and predictable food source during the breeding season [44]. This predictable availability likely contributes to a comparatively narrower isotopic niche at these sites. By contrast, at Cape Möbius and Cape Washington, skuas occasionally exploit alternative prey associated with Weddell seals and Emperor Penguins, potentially resulting in broader isotopic niches. Although a greater number of potential prey categories were considered for Cape Möbius than for Cape Washington (Table 1), this does not necessarily indicate higher prey diversity or availability in the field. At Cape Möbius, extensive fast ice and the distance from large penguin breeding colonies limit access to a single, predictable prey source. Consequently, skuas are likely to exploit a range of opportunistically available resources, which may contribute to the broader isotopic niche observed at this site. In contrast, although fewer potential prey categories were considered for Cape Washington, access to isotopically distinct resources such as Emperor Penguins and marine-derived prey may result in strong trophic contrasts, thereby expanding isotopic niche breadth despite lower apparent prey diversity (Fig 4).

The foraging behaviors observed at each site reflect the ecological context of local prey availability. At Cape Hallett, an Adélie Penguin colony with approximately 40,000 breeding pairs is present, and the adjacent fast sea ice in Edisto Inlet is annually used by breeding Weddell seals. Additionally, approximately 4 km from Cape Hallett, Cape Roget supports a breeding colony of around 9,000 pairs of Emperor Penguins (Fig 5). The surrounding fauna provides skuas with opportunities to access a diverse range of food resources. Nevertheless, the consistent presence of Adélie Penguins suggests that they remain the primary food source, with krill and fish likely accessed through secondary predation. At Cape Washington, a large Emperor Penguin colony is present, although its relatively stable food resources may be less accessible than those associated with Adélie Penguins. The proximity of the site to the open ocean may also increase the likelihood of marine prey consumption. Although the skua breeding site at Cape Möbius is located in close proximity to major penguin colonies—Adélie Cove and Inexpressible Island (Adélie Penguins) and Cape Washington (Emperor Penguins)—skuas at Cape Möbius exhibit a distinct opportunistic foraging strategy. This finding possibly reflects an adaptive response to avoid direct competition with territorial skuas that defend feeding areas around each penguin colony. Instead of engaging in such competition, skuas at Cape Möbius opportunistically exploit penguin-derived resources when available and rely on birth remains and organic material associated with Weddell seals breeding on the adjacent fast ice. By contrast, at Inexpressible Island, skuas show a strong dietary dependence on Adélie Penguins; however, their accessibility to the adjacent marine environment possibly facilitates frequent consumption of krill and fish.

Our findings are consistent with those of previous studies that have documented the opportunistic and regionally variable diet of skuas [3,8,11,45]. Skuas breeding in regions dominated by Adélie Penguins primarily exploit penguin-derived food sources, whereas those breeding in areas with fewer penguins exhibit a more diverse diet, often incorporating marine-derived prey [44,46–48]. Collectively, these findings support the hypothesis that skuas adjust their foraging strategies in response to local prey availability, rather than exhibiting fixed dietary specialization. Furthermore, with the exception of Trillmich (1978) [49], this study represents the only investigation in over four decades into the feeding ecology of the South Polar Skua in Northern Victoria Land, thereby providing valuable and updated insights.

Given the significant ecological role of skuas as scavengers and predators within the Antarctic food web [4,50], the regional dietary differences may carry broader ecological implications [51]. Shifts in prey availability, whether driven by climate change, fluctuations in penguin and seal populations, or anthropogenic pressures such as commercial fishing, have the potential to alter skua foraging strategy and, consequently, isotopic profiles [50].

Although this study provides valuable insights into the dietary ecology of skuas, several limitations should be acknowledged. First, the relatively small sample size may have contributed to bias in isotope values and diet contributions. Given the considerable individual-level variation characteristic of this species, a larger sample size would likely have improved the resolution of regional differences in diet composition. Second, blood samples were collected at intervals throughout the incubation period, which may have resulted in temporal bias. As hatching in this species typically begins in late December [7,52], prey availability is expected to vary over time in response to environmental changes [4,5,7]. Future studies incorporating long-term monitoring and a sufficiently large sample size are expected to yield more robust and comprehensive results. Third, while cannibalism is a well-documented and frequent behavior in skuas [6,11,36], it was not considered in our dietary analysis. In the Cape Möbius region, conspecific predation has been reported to account for the failure of over 50% of eggs to hatch (unpublished data). Thus, the omission of this factor may have introduced errors or underestimation in our interpretation of prey sources.

Stable isotope analysis provides a broad-scale assessment of trophic ecology, and our findings highlight the opportunistic foraging behavior of South Polar Skuas in response to local prey availability. However, this approach lacks the taxonomic resolution to identify specific prey items. Thus, long-term monitoring that integrates complementary methods, such as direct observation, GPS tracking, and DNA metabarcoding, is needed to better understand their foraging ecology and ecological role in a rapidly changing Antarctic environment.

## Supporting information

S1 Fig South Polar Skua (*Stercorarius maccormicki*) feeding on (a), (b) fish; (c) penguin; and (d) seal stomach, as captured by a monitoring camera at Cape Möbius. All photographs were taken by the authors and are original images, published under the CC BY 4.0.
(TIF)

S1 File Source data for table and figures. This file contains the numeric data used to generate the Table 1 and Figure 2–5 presented in the main manuscript.
(XLSX)
