# Supplementary figures and images for: Regional variations in the diet of the South Polar Skua (Stercorarius maccormicki) in the Ross Sea region, Antarctica
Source: PLoS One. 2026 Feb 9;21(2):e0341112. doi: 10.1371/journal.pone.0341112 (PMC12885291; doi:10.1371/journal.pone.0341112)

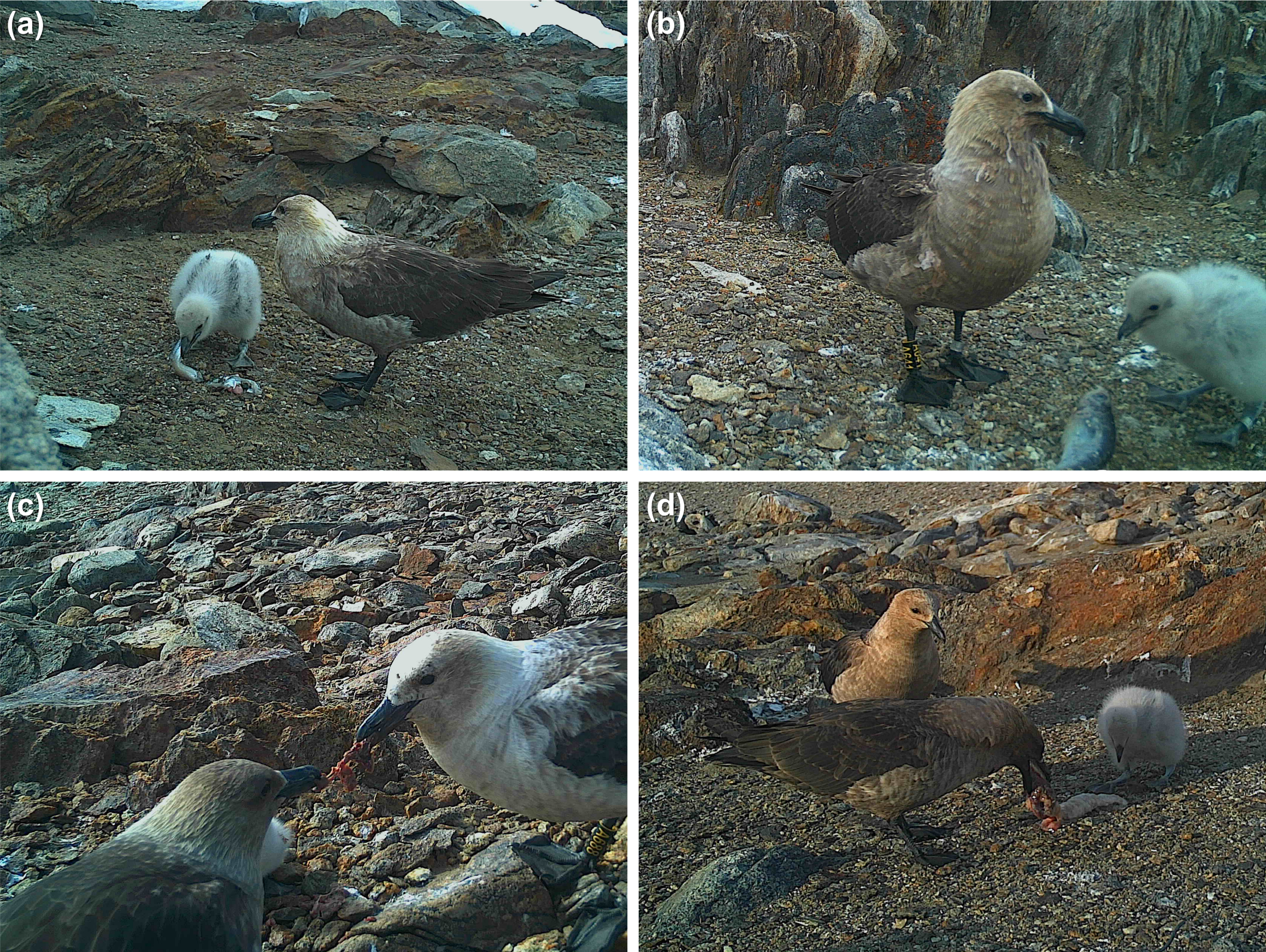

Supplement: S1 Fig — All photographs were taken by the authors and are original images, published under the CC BY 4.0. (TIF) [file pone.0341112.s001.tif]
